# Overcoming multidrug resistance: antimicrobial peptides as a next-generation therapeutic strategy

**DOI:** 10.3389/fcimb.2026.1745427

**Published:** 2026-02-19

**Authors:** Shiqi Zhou, Zixuan Sun, Luojia Liu, Yuanyin Xi, Linxi Zhou, Zhibing Yang, Junli Zhou

**Affiliations:** 1 Department of Burn and Plastic Surgery, The Tenth Affiliated Hospital, Southern Medical University (Dongguan People’s Hospital), Dongguan, Guangdong, China; 2 Department of Plastic, Reconstructive and Cosmetic Surgery, Xinqiao Hospital, Army Medical University, Chongqing, China; 3 Department of Orthodontics, College & Hospital of Stomatology, Guangxi Medical University, Nanning, Guangxi, China; 4 Department of Plastic Aesthetic Surgery, State Key Laboratory of Trauma, Burns and Combined Injury, Southwest Hospital, Army Medical University, Chongqing, China; 5 Department of Breast and Thyroid Surgery, Southwest Hospital, Army Medical University, Chongqing, China

**Keywords:** antibacterial peptides, antibiotic drug resistance, clinical transformation, mechanisms of action, membrane bioactive peptides, redesign and modify

## Abstract

Amidst the escalating global threat of antibiotic resistance, there is an urgent need for novel antibacterial agents with distinct mechanisms of action to address the impending post-antibiotic era. Antimicrobial peptides (AMPs), membrane-active peptides characterized by rapid bactericidal effects, broad-spectrum activity, and low resistance development potential, are considered promising candidates for overcoming the current multidrug resistance (MDR) crisis. However, the clinical application of AMPs is significantly limited by inherent drawbacks, including susceptibility to proteolytic degradation, poor oral bioavailability, potential mammalian cytotoxicity, low *in vivo* efficacy, and high production costs. These limitations have spurred extensive efforts to redesign and modify AMPs based on their physicochemical properties and mechanisms. This review first summarizes four classical models describing the initial binding and membrane disruption processes of AMPs. It then focuses on recent advancements in the chemical synthesis and modification of AMPs, as well as AMP-based drug delivery systems. In conclusion, this review provides a comprehensive perspective on the mechanisms of action, progress in novel therapeutic strategies, and prospects for the clinical translation of AMPs.

## 1 Introduction

Infectious diseases remain a persistent global health burden, accounting for >20% of annual deaths worldwide. The increasing prevalence of invasive medical procedures (e.g., organ transplantation, chemotherapy), immunosuppressive therapies, and aging populations has significantly expanded cohorts vulnerable to bacterial pathogens. Surgical site infections (SSIs) alone affect 11% of patients in developing countries, prolonging hospitalization by around 8 days and increasing mortality by several folds, incurring approximately 3.5billionto10 billion in US healthcare costs annually (Jara et al., 2019; Atherton et al., 2025). In intensive care units, ventilator-associated pneumonia (VAP) impacts 9-27% of intubated patients, with attributable mortality reaching 50% in multidrug-resistant (MDR) cases (Hammond et al., 2022). These realities underscore the critical necessity for effective antimicrobial interventions across modern healthcare.

Statistical modeling estimates that over four million deaths were associated with antimicrobial resistance (AMR) globally in 2019, with more than one million deaths directly attributable to AMR (Global burden of bacterial antimicrobial resistance in 2019: a systematic analysis, 2022; Li et al., 2025). Furthermore, the 2016 Review on Antimicrobial Resistance projected that by 2050, up to 10 million people could die annually from AMR (Atherton et al., 2025). Consequently, developing new antibacterial agents targeting antibiotic-resistant bacteria represents an urgent challenge in clinical treatment. However, the WHO reports “Antibacterial Agents in Clinical and Preclinical Development” (2020, 2021) indicate that the development pipeline for alternative drugs remains largely stagnant (2021 Antibacterial agents in clinical and preclinical development an overview and analysis).

Most conventional antibiotics function by repressing or killing target bacteria through site-specific binding mechanisms. This approach inherently faces the risk of resistance development due to mutations in binding sites or structural alterations in the antibiotics themselves (Pang et al., 2019; Wencewicz, 2019).Additionally, since many antibiotics act intracellularly, reduced membrane permeability and enhanced efflux pump activity constitute major contributors to resistance (Pang et al., 2019; Tam et al., 2021). Therefore, despite some debate regarding the aforementioned projections, the pressing need for antibacterial agents employing distinct mechanisms to combat the advent of the post-antibiotic era is undeniable.

AMPs, membrane-active peptides widely present in eukaryotic multicellular organisms, were first discovered in the mid-1990s following bacterial or fungal infection of *Drosophila melanogaster (*Rodríguez-Rojas et al., 2014; Gan et al., 2021; Maglangit et al., 2021). For the purposes of this review, which focuses on membrane disruption, AMPs are broadly defined as: charged or uncharged bioactive peptides, typically 2–100 amino acid residues in length, derived from diverse sources and exhibiting varied structures. They possess either direct antimicrobial activity or indirect activity by potentiating the effects of other drugs (Gan et al., 2021; Schafer et al., 2021). AMPs can be produced by cellular machinery, chemical synthesis, or a combination of both (Chaudhuri et al., 2021; Li et al., 2021). AMPs were traditionally considered broad-spectrum, rapidly acting, and less prone to resistance development (Magana et al., 2020), however, recent evidence highlights unexpected specificity and high synergistic potential, further enhancing their clinical promise (Lazzaro et al., 2020).

Understanding the mechanism of AMP action is essential for the rational design and development of new AMP therapies. AMPs were initially defined as membrane-active peptides exerting antibacterial effects primarily through membrane disruption, a mechanism detailed later (Chai et al., 2019; Wang et al., 2019). However, ongoing research has revealed increasingly diverse antibacterial mechanisms. For instance, human defensin HD5, secreted by intestinal Paneth cells, contributes to innate host defense against intestinal pathogens and shapes a protective neonatal gut microbiota against pancreatic autoimmunity (Yang and Shen, 2021; Fu et al., 2022; Liang et al., 2022). HD5 can sequester pathogens from the host intestinal epithelium, forming a chemical barrier to limit tissue inflammation and microbial translocation. It can also bind to the ligand-binding domain (LBD) of ACE2, inhibiting SARS-CoV-2 invasion (Wang et al., 2020).

In such immune-modulating contexts, AMPs are also referred to as host defense peptides (HDPs) (Becker et al., 2010; Gao et al., 2021). However, HDPs can sometimes act as a double-edged sword. Excessive production of LL-37 may contribute to pseudoanaphylaxis, allergic contact dermatitis, urticaria, and mast cell hyperplasia (Harder et al., 2010; Niyonsaba et al., 2010; Muto et al., 2014). Conversely, *Shigella* can exploit HD5 to enhance its adhesion and invasion capabilities (Xu et al., 2018). AMPs can also combat infection by inhibiting bacterial biosynthesis. For example, LL-37 and magainin-2 form pores in the membranes of *S. aureus* and *E. coli*, facilitating the entry of histone H2A into the cytoplasm. Inside the cell, H2A reorganizes bacterial chromosomal DNA and inhibits global transcription (Doolin et al., 2020). Some AMPs may also impede bacterial attachment to surfaces by inhibiting swimming or swarming motility or interfering with flagellar assembly (Hell et al., 2010; de la Fuente-Núñez et al., 2012).

In summary, the primary mechanisms of AMP include: (a) direct antimicrobial activity via membrane disruption; (b) indirect antimicrobial activity through immune modulation; (c) inhibition of bacterial biosynthetic functions, as in **Table 1**. Unlike traditional antibiotics that require entry into bacteria and targeting specific targets such as protein or nucleic acid synthesis, membrane disruption mechanism is a direct physical attack. AMPs are electrostatically adsorbed onto bacterial membranes, disrupting their physical integrity and causing leakage of cellular contents and bacterial death. This mode of action is like a ‘physical explosion’, with extremely fast speed, making it difficult for bacteria to cope and develop resistance through conventional biochemical reactions. Therefore, this review starts from the limitations, then primarily focuses on the membrane-disrupting effects of AMPs, and on this basis, also introduces recent modification and targeted delivery strategies in the development of AMPs. Recently, many reviews on AMPs have been published, but some focused on certain pathogens or diseases (Owliaee et al., 2025; Zhang, 2025), while others only introduce optimization strategies for AMP (Wu et al., 2025). This review also summarizes and introduces the targeted delivery strategies with clinical application prospects.

**Table 1:** Examples of AMPs and the corresponding models of membrane.

Name of antimicrobial peptides (AMPs)	Source	Main membrane disruption model	Key characteristics
Trematocine	Antarctic fish	carpet model	α-helical conformation, reduced membrane fluidity
(P)GKY20	Unknown	carpet model	Low hemolytic activity and significant efficacy against Gram-negative bacteria
Megin	Shovel-footed toad	carpet model	Located at the lipid-water interface,interfering with lipid order
Protegrin-1(PG-1)	Porcine leukocytes	barrel-stave model/electroporation model	High charge density, capable of inducing molecular electroporation
Magainin-2	Xenopus laevis	toroidal-pore model	Form transmembrane pores
Melittin	Bee Venom	toroidal-pore model	Form transmembrane pores
Aurein1.2	Australian fire-bellied toad	Unknown	The activity was enhanced after the substitution of Asp4 and Glu11 residues.

## 2 Limitations and challenges of AMPs

AMPs are considered a new generation of candidate drugs for addressing the global antibiotic resistance crisis due to their broad-spectrum antibacterial activity, low resistance induction, and immune regulation advantages. However, its inherent defects seriously hinder the clinical translation process, among which the three most prominent core limitations are insufficient protease sensitivity and *in vivo* stability, imbalance between toxicity and host selectivity, disconnection between *in vivo* and *in vitro* activity, and low bioavailability, and there are significant causal relationships and mechanism coupling between each limitation.

Natural AMPs are mostly linear sequences of L-amino acids, which are easily degraded by proteases from both the host and pathogenic microorganisms. They are cleared quickly by the liver and kidneys, have a very short half-life in the body, and are difficult to maintain effective therapeutic concentrations. From the perspective of degradation sources, trypsin at the host level can rapidly hydrolyze natural AMPs, with most L-amino acid AMPs degrading over 50% in human plasma within 4 hours; At the pathogen level, pathogenic bacteria can secrete proteases to target the degradation of AMP, weakening their antibacterial activity (Kumar et al., 2019; Lu et al., 2020). Although chemical modification can improve stability, there are still limitations: although AMP modified with all D-amino acids has increased resistance to trypsin degradation, it will reduce antibacterial activity and cause acute *in vivo* toxicity due to conformational changes from alpha helix to random coil; Introducing only N-terminal α-aminoisobutyric acid modified AMP can enhance plasma stability, but cannot completely resist serum protease degradation (Kumar et al., 2019; Neubauer et al., 2020).

In addition to stability defects caused by protease sensitivity, AMPs also have core limitations of toxicity and host selectivity imbalance. Its antibacterial activity depends on cationic properties and a balance between hydrophilicity and hydrophobicity: a high net positive charge enhances non-specific binding to the host cell membrane, while a high hydrophobicity reduces bacterial membrane targeting. The imbalance between the two is the fundamental cause of off target toxicity (Kumar et al., 2019; Lu et al., 2020).

Besides, the disconnection of *in vitro* and *in vivo* activity and extremely low oral bioavailability further hinder their clinical translation. At the active level, many modified AMPs exhibit excellent antibacterial activity *in vitro*, but their efficacy *in vivo* is significantly reduced; At the level of bioavailability, natural AMPs are not only easily hydrolyzed by gastrointestinal proteases after oral administration, but also unable to effectively penetrate the intestinal epithelial barrier due to the imbalance of hydrophilicity and hydrophobicity; In addition, it will be quickly cleared by the liver and kidneys, resulting in extremely short circulation time in the body; Even if the half-life is prolonged by modification, its pharmacokinetic parameters are still far inferior to commonly used antibiotics in clinical practice. Meanwhile, AMPs with strong hydrophobicity can also aggregate on the surface of intestinal mucosa, which further hinders their cross epithelial absorption (Lu et al., 2020; Ortiz-Gómez et al., 2020).

Last but not least, high production costs are another core obstacle to the large-scale clinical application of AMPs. The chemical synthesis process of AMPs is complex, especially in the synthesis of long-chain peptides and modified peptides, where raw material loss and purification difficulty are high, resulting in significantly higher unit production costs than traditional antibiotics. Although the development of synthetic biology technology has provided a new pathway for the recombinant expression of AMP, such as using E. coli to construct a microbial factory that can reduce production costs by about 60%, recombinant expression still faces problems such as low expression levels and easy degradation of products (Gunasekera et al., 2020). For therapeutic scenarios that require high-dose administration, high production costs can directly affect drug accessibility, and this challenge needs to be addressed through further breakthroughs in process optimization and synthetic biology technology.

## 3 Mechanisms of membrane disruption

The direct membrane-disrupting effect of AMPs can be divided into two stages. The first stage involves the initial docking of the AMP onto the bacterial membrane, primarily dependent on the compositional differences between host and pathogen membranes, conferring selectivity. Mammalian cell surfaces typically consist largely of neutral phospholipids, such as sphingolipids or phosphatidylcholine. In contrast, bacterial membranes often contain a significant proportion of negatively charged phospholipids, including phosphatidylserine (PS), cardiolipin, and phosphatidylglycerol (PG) (Matsuzaki, 2009; Knirel, 2011; Tang and Sails, 2014). The overall negative charge of bacterial membranes is likely a key evolutionary driver for the predominantly cationic nature of most AMPs. Initial binding is facilitated by electrostatic attraction between the cationic AMP and the anionic bacterial surface. Previous evidence suggests that increasing the net positive charge correlates with enhanced antimicrobial activity but can also increase off-target toxicity beyond a certain threshold (Yeaman and Yount, 2003; Jiang et al., 2008; Gawde et al., 2022). Most AMPs are amphipathic, typically exhibiting ~50% hydrophobicity. Increased hydrophobicity has been shown to improve antibacterial activity. However, as both mammalian and bacterial cell membranes possess hydrophobic cores, elevated AMP hydrophobicity can reduce bacterial selectivity, potentially leading to hemolysis. Furthermore, excessive hydrophobicity in AMP aggregates can diminish antimicrobial activity (Chen et al., 2007; Xiong et al., 2017).Therefore, maintaining a balance in both net charge and hydrophilicity/hydrophobicity is a prerequisite for the potential clinical application of AMPs. In addition, in addition to negatively charged phospholipids such as PG and cardiolipin, bacterial membranes also contain unique lipid components: lipopolysaccharides (LPS) in the outer membrane of Gram negative bacteria, and phosphatidic acids (TA) in the cell wall of Gram positive bacteria. For example, tryptophan (Trp) residues in AMP can be embedded into the lipid A region of LPS through hydrophobic interactions, while arginine (Arg) can form hydrogen bonds with the phosphate group of TA. This precise matching of “lipid amino acid residues” can further enhance selectivity (Wang et al., 2020).

### 3.1 Toroidal-pore model

The second stage involves bacterial killing following initial binding, for which several models exist. We summarize four major models: the toroidal-pore, barrel-stave, carpet, and electroporation models. In both the barrel-stave and toroidal pore models, hydrophobic residues of the AMPs interact with the membrane core, while hydrophilic residues form a transmembrane polar channel. Lipid headgroups are typically present within this channel, with peptides arranged more loosely in toroidal pores (Jenssen et al., 2006; Wimley, 2010; Gan et al., 2021).

**Figure 1:**
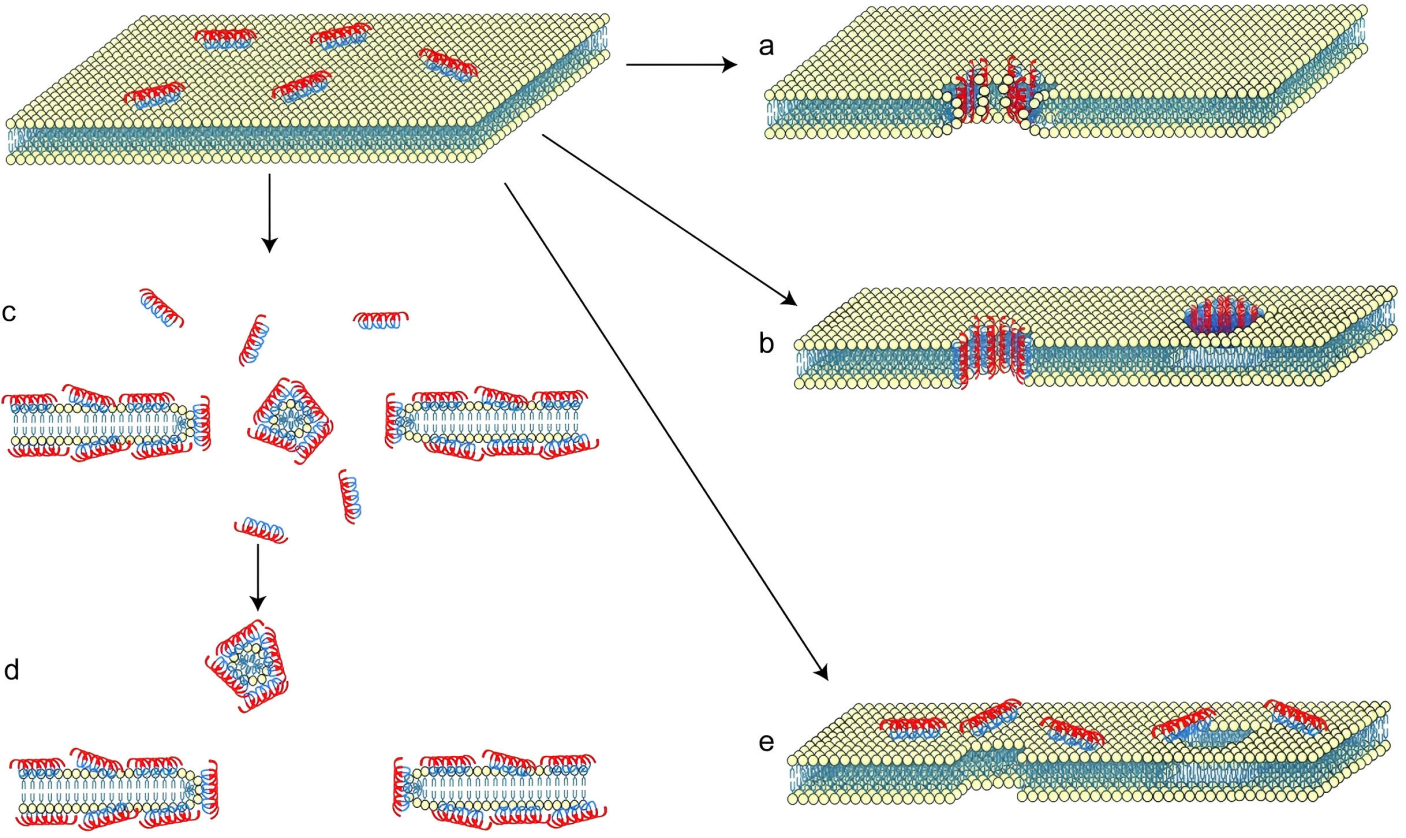
Membrane destruction mechanism of AMPs. **(A)** The toroidal pore model. **(B)** The barrel-stave pore model. **(C)** The carpet model. **(D)** The detergent model. **(E)** The electroporation model. Figure was modified with copyright permission from Bee Ha Gan et al. and Jasmin Portelinha et al (Lee et al., 2005).

The toroidal-pore model proposes that peptides interacting vertically with the membrane in a non-transmembrane manner induce membrane curvature. This curvature separates polar head groups from lipid tails, ultimately disrupting membrane integrity (**Figure 1a**) (Lee et al., 2005). The critical packing parameter (CPP), initially proposed by Israelachvili et al., describes lipid cluster geometry as a function of the surface area (a0), the hydrophobic lipid chain length (l) and lipid volume (v) such that the CPP = v/a0l (Israelachvili et al., 1980). Building on the toroidal-pore model and CPP, Thomas Berry and colleagues suggested that peptide insertion decreases the weighted CPP (CPPw), promoting lipid diffusion into curved pore regions and increasing pore diameter. The research data shows that the CPPw of bacterial membrane lipids decreases from 0.8 to 0.5 after peptide insertion, and this decrease is positively correlated with the expansion of pore size from about 1.2 nanometers to 2.5 nanometers, confirming the direct correlation between lipid accumulation disturbance and pore formation. This indicates that peptide interactions influence the effective hydrated headgroup area of neighboring lipids and disrupt lipid packing within the bilayer (Berry et al., 2018).

In addition, the stability and size of the cyclic pore are further regulated by the structural characteristics of AMP, especially the twisting of proline or glycine in the helical peptide. Proline conjugation can limit the flexibility of peptide chains, anchor the circular pore structure, and maintain its diameter stably at 2-3nm to ensure effective leakage of cellular contents; In contrast, glycine twisting increases the flexibility of the peptide chain, leading to the collapse of the circular pore structure and loss of membrane breaking activity (Tuerkova et al., 2020). Molecular dynamics simulations provide direct conformational evidence for the formation process of circular pores in typical AMPs such as Pin2. Specifically, the α- helix region (residues 5-12) of Pin2 is inserted into the hydrophobic core of the bacterial membrane, while its hydrophilic region (residues 1–4 and 13-18) forms the inner wall of the circular pore. Polar amino acid side chains (such as serine and asparagine) interact with lipid head groups and water molecules to stabilize the pore channel (Velasco-Bolom and Garduño-Juárez, 2022). The conformational feature of Pin2 further confirms its preference for the circular pore model over other transmembrane pore models.

### 3.2 Barrel-stave model

The barrel-stave model describes peptides reaching a threshold concentration, forming a bundle with a central lumen that inserts into the membrane, resembling a barrel-shaped transmembrane pore primarily composed of helical peptides (**Figure 1b**). Unlike the non transmembrane curved structure of the annular pore model, the bucket wall pore requires AMP to completely span the membrane bilayer, where the hydrophobic surface of the helical peptide aggregates to form the outer wall of the pore (interacting with the membrane lipid tail), and the hydrophilic surface faces the central cavity (forming a permeable channel) (Jenssen et al., 2006). It is worth noting that the regulation of spiral bending on the barrel-stave noodles model is diametrically opposite to the annulus model. The research shows that proline bending can stabilize the annular pore structure by limiting the flexibility of peptide chains, but it will destroy the orderly assembly of helical peptide bundles required for the formation of barrel-stave noodles pores - because it will break the continuous hydrophobic interface between adjacent peptide chains; Glycine bending will further exacerbate this damage by increasing the mobility of the peptide chain, ultimately leading to the disintegration of the barrel like structure and the inability to form transmembrane pores. This difference in regulation mediated by bending is the core structural basis that distinguishes the two models (Tuerkova et al., 2020). There is an interesting controversial case about the specificity of the pore forming model, whose research object is AMPPin2: the early hypothesis believed that Pin2 may form a barrel-stave noodles pore due to its α - helical structure, but the molecular dynamics simulation of Jose Luis Velasco Colom showed that Pin2 antibacterial peptide formed an annular pore that allows water molecules to pass through, rather than a barrel-stave noodles pore model (Velasco-Bolom and Garduño-Juárez, 2022). The research shows that proline bending can stabilize the annular pore structure by limiting the flexibility of peptide chains, but it will destroy the orderly assembly of helical peptide bundles required for the formation of barrel-stave noodles pores - because it will break the continuous hydrophobic interface between adjacent peptide chains; Glycine bending will further exacerbate this damage by increasing the mobility of the peptide chain, ultimately leading to the disintegration of the barrel like structure and the inability to form transmembrane pores. This difference in regulation mediated by bending is the core structural basis that distinguishes the two models (Tuerkova et al., 2020). There is an interesting controversial case about the specificity of the pore forming model, whose research object is AMPPin2: the early hypothesis believed that Pin2 may form a barrel-stave noodles pore due to its α - helical structure, but the molecular dynamics simulation of Jose Luis Velasco Colom showed that Pin2 antibacterial peptide formed an annular pore that allows water molecules to pass through, rather than a barrel-stave noodles pore model (Velasco-Bolom and Garduño-Juárez, 2022).

### 3.3 Carpet model

The carpet model illustrates peptides accumulating parallel to the membrane surface like a carpet, eventually causing disintegration and formation of peptide-wrapped membrane fragments (**Figure 1c**). The latter stage resembles detergent action, hence the alternative name “detergent” model (**Figure 1d**). This occurs when peptide density on the membrane reaches a high peptide-to-lipid ratio. Peptides spontaneously arrange parallel to the surface, with hydrophilic sides facing lipid headgroups and hydrophobic portions oriented towards lipid tails. Membrane disruption requires reaching a critical threshold peptide density (peptide-to-lipid ratio typically between 0.1% and 1%). Once achieved, this accumulation compromises structural integrity, leading to massive leakage of cellular contents. Trematocine, a novel AMP discovered in an Antarctic fish, folds into an α-helical conformation upon interaction with vesicles bearing zwitterionic or anionic charges. Giulia Della Pelle reported a highly specific interaction between Trematocine and anionic vesicles. Studies using the fluorescent probe 1,6-Diphenyl-1,3,5-hexatriene (DPH), which reports on membrane microviscosity (reduced anisotropy indicates looser acyl tail packing), revealed that DPH anisotropy increasedin anionic large unilamellar vesicles (LUVs) upon Trematocine addition. This suggests a novel bilayer perturbation mechanism that decreasesmembrane fluidity, similar to effects observed with magainin 2 and melittin (Della Pelle et al., 2020). (P)GKY20, an AMP with low eukaryotic hemolytic activity but high efficacy against *Gram-negative bacteria*, was characterized by Rosario Oliva and colleagues. Their experimental results align with a carpet-like mechanism: (i) initial electrostatic recruitment of peptides to anionic lipids induces helical folding; (ii) formation of large peptide-anionic lipid domains induces positive membrane curvature; (iii) upon reaching a threshold, peptide-mediated lipid interactions extract bilayer lipids, compromising membrane integrity (Oliva et al., 2019). Megin, an AMP from a spadefoot toad, exhibits good antibacterial activity. L.R. Bogdanova and colleagues used molecular dynamics (MD) simulations, zeta potential measurements, and other techniques to explore megin’s interaction with model biomembranes. Evidence indicated that megin predominantly resides at the lipid-water interface, arranged parallel to the bilayer surface like a carpet. MD simulations confirmed that peptide insertion disturbs lipid ordering (decreased order parameters), facilitating water penetration and leakage of cellular contents (Bogdanova et al., 2020).

### 3.4 Electroporation model

The electroporation model proposes that charged peptides accumulate on the outer membrane surface, generating a sufficient potential difference to induce pore formation (**Figure 1e**). Unlike other models, the pores and peptides are relatively independent in this model. The number of pores does not increase with external pressure; instead, the key parameter is the mechanical stress difference between the outer and inner membrane leaflets (Leontiadou et al., 2006). Given the charge carried by most AMPs, an electric field is invariably induced on the membrane surface. Therefore, mechanical stress and electroporation may jointly underlie AMP-induced pore formation. Supporting this hypothesis, Frantz Jean-François and colleagues presented evidence (numerical simulations, electrophysiological measurements, NMR-derived data) suggesting minimal bilayer perturbation by the peptides, consistent with an electroporation mechanism (Jean-François et al., 2008). Protegrin-1 (PG-1), a cationic AMP rich in arginine, is generally thought to disrupt membranes via transmembrane pore formation. Pin-Kuang Lai et al. employed MD simulations to investigate the role of bidentate complexes in PG-1 translocation and utilized computational electroporation methods. Their observations indicated that PG-1 integrates into the membrane, and an applied external electric potential facilitates the formation of a water-permeable column within the bilayer. PG-1, with its high charge density, was found to induce molecular electroporation. This hypothesis was further supported by experiments substituting arginines with equally charged lysines, which significantly reduced insertion efficiency and pore-forming ability (Lai and Kaznessis, 2018).

## 4 The immune regulatory function of AMP

AMPs can not only achieve direct sterilization through membrane disruption, but also participate in anti-infection and immune homeostasis maintenance by regulating the host immune system in multiple dimensions. Their immune regulatory effects cover the entire chain of innate and adaptive immunity, and have significant functionality and limited applications. Therefore, it is crucial to search for compounds that selectively affect the target and develop appropriate application methods (Owliaee et al., 2025).

### 4.1 Bi directional regulation of inflammatory response

Antimicrobial peptides, as key regulatory factors of host immune homeostasis, are an important bridge connecting innate immunity and adaptive immunity. In addition to recruiting immune cells, enhancing antigen presentation, and regulating lymphocyte polarization, the bidirectional regulation of inflammatory response is the core of AMPs’ immune regulatory function - this characteristic enables antimicrobial peptides to quickly initiate antimicrobial defense in the early stages of infection and suppress excessive inflammation to avoid tissue damage. Its mechanism of action is closely related to specific signaling pathways, tissue microenvironments, and peptide structure characteristics, and this conclusion has been validated in relevant studies on lung, dental pulp, and systemic mucosal tissues.

#### 4.1.1 Inflammatory regulation: initiation of immune response in the early stages of infection/injury

In the early stages of pathogen invasion or tissue damage, the core role of antimicrobial peptides is to activate the pro-inflammatory signaling network, recruit innate immune cells, initiate inflammatory cascade reactions, and build the first line of defense for rapid clearance of pathogens. The activation of this function requires three conditions to be met: first, a high pathogen load in the early stages of infection, which provides a triggering factor for pro-inflammatory signals; Secondly, the peptide segment is in the “pro-inflammatory threshold” range, such as 1–10 nM for HNPs and>10 μ g/mL for LL-37. If it is below this threshold, the pathway cannot be effectively activated; Thirdly, the organizational microenvironment is dominated by pathogen invasion, and there is no obvious self-tissue damage (Owliaee et al., 2025; Wu et al., 2025). After meeting the above conditions, the completion of inflammatory response still depends on precise receptor binding and pathway activation: high concentrations of cat antimicrobial peptide LL-37 in the lungs can specifically bind to FPR2 receptors on the surface of macrophages to secrete pro-inflammatory factors such as tumor necrosis factor alpha (TNF - α) and interleukin-1 β, ultimately completing early defense against pulmonary infection. The metabolic products of gut microbiota, Doreamides (antimicrobial peptide like substances), can directly activate the NLRP3 inflammasome of immune cells, induce the release of IL-1 β, and trigger the secretion of endogenous antimicrobial peptide HD5 by host epithelial cells, forming a cascade pro-inflammatory mechanism of “microbiota metabolites antimicrobial peptides immune clearance”, enhancing the early anti-infective ability of the gut (Wu et al., 2025).

#### 4.1.2 Anti-inflammatory regulation: stop loss of damage caused by excessive inflammation

When the inflammatory response exceeds the physiological regulation range and begins to threaten the integrity of the host tissue, antimicrobial peptides quickly switch to an anti-inflammatory phenotype, achieving “inflammation stop loss” by blocking pro-inflammatory pathways, inducing anti-inflammatory factor secretion, repairing tissue barriers, and other methods. Their effects also depend on specific targets and pathways, such as the AhR pathway. Low concentrations (<5 μ g/mL) of LL-37 in the lungs can activate macrophage AhR receptors, induce anti-inflammatory factor IL-10 secretion, reduce the inflammatory infiltration area of burn wounds by 32%, and achieve inflammation resolution and tissue protection in the lungs and surface injury sites (Wu et al., 2025) At the same time, antimicrobial peptides from different tissues will activate differentiated anti-inflammatory mechanisms based on their own structural and functional characteristics: the combination of IDR-1002 and ciprofloxacin in dental pulp tissue can significantly downregulate the expression of TNF - α and IL-6 by regulating the inflammatory cytokine spectrum, while upregulating IL-10 levels, achieving a synergistic effect of “anti-inflammatory+antibacterial” and providing a stable immune microenvironment for dental pulp regeneration (98). Similar to early pro-inflammatory regulation, the activation of AMPs’ anti-inflammatory function also requires three core conditions: first, the inflammation enters the middle and late stages, and there is a potential risk of tissue damage; Secondly, if the peptide concentration exceeds the pro-inflammatory threshold, such as LL-37<5 μ g/mL, the high concentration pro-inflammatory phenotype will switch to the low concentration anti-inflammatory phenotype; Thirdly, the organizational microenvironment has shifted from being dominated by pathogen invasion to being dominated by tissue damage, requiring the initiation of anti-inflammatory programs to protect one’s own tissues (Wu et al., 2025,98).

### 4.2 Structural basis of bidirectional regulation: synergistic effect of concentration gradient, peptide conformation, and tissue microenvironment

The bidirectional regulation of inflammation by antimicrobial peptides is a precise balance between their own characteristics and the synergistic effect of tissue microenvironment, which relies on three major foundations. The concentration gradient is the core switch, for example, HNPs are pro-inflammatory at 1-10nM and anti-inflammatory at 10-100 μ M, and the lung LL-37 concentration threshold is regulated by the airway environment, with low concentration anti-inflammatory and high concentration pro-inflammatory. The peptide structure is the molecular basis, and the net positive charge, hydrophobic conformation, and disulfide bond affect its signal initiation and anti-inflammatory activity, respectively. The organizational microenvironment determines the adaptability of the scene. The acidic environment of the CF airway inhibits the anti-inflammatory activity of PLUNC, while the highly mineralized microenvironment of the dental pulp can reshape the function of hBD-4, making it both anti-inflammatory and promoting stem cell differentiation.

## 5 Progress in new AMP therapeutic strategies

**Figure 2:**
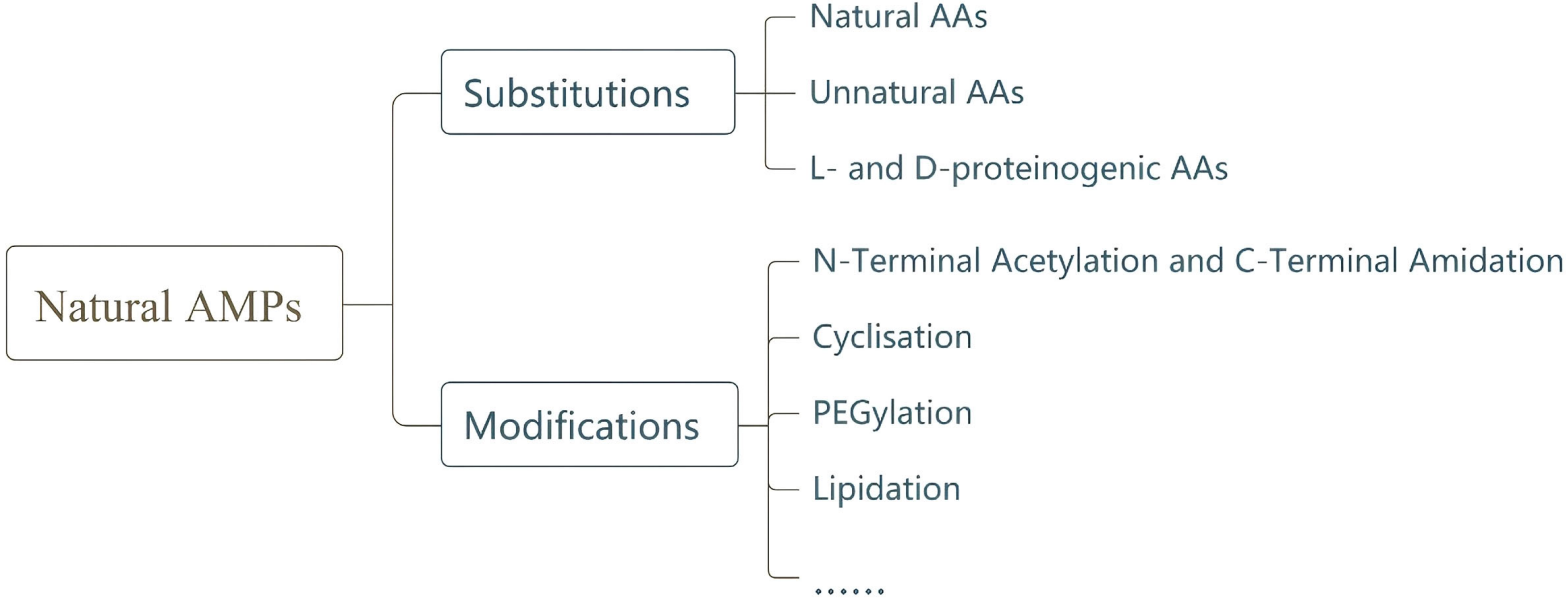
Chemical synthesis and modifications of AMPs.

Despite promising antibiotic properties and low resistance development potential, AMPs possess inherent defects that limit their clinical application, including susceptibility to proteolytic degradation, poor oral bioavailability, potential mammalian cytotoxicity, low *in vivo* efficacy, and high production costs (Torres et al., 2019; Schafer et al., 2021; Ngambenjawong et al., 2022). To overcome these challenges, researchers have modified natural AMPs based on their mechanisms of action. This section reviews recent improvements in AMP optimization strategies (**Figure 2**).

### 5.1 Substitution of natural amino acids

For clinical use, AMPs must exhibit sufficient *in vivo* stability, potent antibacterial efficacy, and minimal biological toxicity. These properties are largely governed by their biochemical selectivity for negatively charged microbial membranes. Antimicrobial activity critically depends on a balanced interplay of charged residues, hydrophobicity, amphipathicity, and secondary structure (e.g., α-helix, β-sheet) (Yadav and Chauhan, 2024). Cytotoxicity, however, stems from hydrophobic interactions between residues (e.g., Trp, Leu, Tyr) and zwitterionic phospholipids on host cell surfaces. Pseudin-2, isolated from Pseudis paradoxa, exhibits potent bactericidal activity but also cytotoxicity.

Pseudodin-2 isolated from *Pseudodis paradoxa* exhibits potent bactericidal activity against multidrug-resistant *Pseudomonas aeruginosa* (MDR-PA), with a minimum inhibitory concentration (MIC) of 16 μ g/mL; However, due to a hemolysis rate of 12.3% and a drug resistance mutation frequency of 1.2 × 10 ^-8^ at a concentration of 1 × MIC, its clinical application is limited (Kang et al., 2018). Recent studies have enhanced the amphiphilic helical structure and cationic properties of Pseudodin-2 analogs by replacing lysine (Lys) residues. Shortened analogues and peptides that form linear 18 residue amphiphilic helices after lysine substitution not only significantly reduce cytotoxicity (hemolysis rate at 1 fold minimum inhibitory concentration (MIC) is only 2.1%), but also enhance antibacterial activity (MIC to 4 μ g/mL for MDR-PA), and the frequency of drug-resistant mutations is reduced to<5.0 × 10 ^-9^ (Kang et al., 2018).

The study by Mauricio Arias and colleagues showed that changing the side chain length of lysine and arginine residues can enhance the resistance of antimicrobial peptides to proteases. This method not only improves the stability of antimicrobial peptides in trypsin digestion, but also maintains or even enhances their antimicrobial activity in some cases. Their research results indicate that in addition to simple arginine lysine substitution, modifying the side chain length is also an effective strategy to improve the stability and activity of antimicrobial peptides (Arias et al., 2018).

### 5.2 Substitution of unnatural AAs

LL-37 (cathelicidin family antimicrobial peptide) has strong anti staphylococcal activity, but is easily degraded by Staphylococcus aureus protease (Yuan et al., 2025). Non proteinogenic amino acids (i.e. non natural amino acids) provide a solution to this problem by breaking through the structural limitations of natural amino acids and achieving precise regulation of antimicrobial peptide (AMP) charge distribution, conformational stability, and enzymatic resistance. It is the core strategy to address the inherent defects of AMP (Lu et al., 2020). From a typological perspective, this strategy covers four major categories of non natural amino acids: firstly, chiral isomers, represented by D-lysine and D-arginine, which enhance stability by disrupting protease L-amino acid recognition sites; secondly, charge regulated types, such as L-2,4-diaminobutyric acid (Dab), L-2,3-diaminopropionic acid (Dap), and L-arginine (Hor), which balance antibacterial activity and cytotoxicity by adjusting the length of the side chain carbon chain. The third type is conformational restriction, including α - aminoisobutyric acid (Aib), cyclopropylalanine (cPro), etc., which can lock the active conformation of peptide chain α - helix/β - folding and reduce the flexibility of peptide bonds (Li et al., 2021; He et al., 2022). The fourth type is functionalized with functional groups, typically α - (4-pentenyl) - alanine and L-thiophene alanine (Thi), which can provide subsequent lipidation and targeted ligand coupling sites.

Compared to AMPs with natural amino acid sequences, the advantages of non natural amino acid substitution exhibit multidimensional scalability: in addition to resistance to protease degradation and enhanced conformational stability, it can also improve pharmacokinetic properties. Jianguang Lu and colleagues developed derivatives of Pep05 by replacing the L-amino acid residues in the cationic antimicrobial peptide Pep05 with non natural amino acids (Dab, Dap, L-thiophenalanine, 4-aminobutyric acid (Aib)). It is worth noting that the introduction of a single Aib at the N-terminus of derivative UP09 significantly prolongs its plasma half-life, with a residual of over 30% at 12 hours, which is much higher than the rapid degradation of natural peptide Pep05 (Lu et al., 2020); The cationic alpha helical antimicrobial peptide Feleucin-K3 from the oriental bell toad was modified by synthesizing its analogues. As a result, it was found that replacing the specific site with α - (4-pentenyl) - alanine (α - (4-Pentenyl) - Ala) significantly enhanced its activity (MIC decreased to 2μM), improved its stability, and the frequency of drug-resistant mutations was less than 1.0×10^-9^. There was no significant hemolysis or resistance observed at antibacterial concentrations (Guo et al., 2021).

### 5.3 Substitution of L- and D-proteinogenic AAs

As charge and amphipathicity are crucial for AMP selectivity and activity, substituting multiple acidic residues with other proteogenic residues, or replacing neutral/anionic AAs with cationic hydrophobic residues, can significantly enhance the antibacterial potency of analogues. Alanine scanning is a widely used method for systematic residue substitution. Each amino acid is individually replaced with Ala, allowing elucidation of the functional role of each residue and providing detailed insights into AMP structure-activity relationships (SAR) (Holman et al., 2021). Staphylococcal δ-toxin-inspired peptides (STIPs) represent a potential source of *anti-MRSA* AMPs. Researchers screened a variant δ-toxin sequence using a pathogen-specific protease management strategy and optimized it through rational design, including alanine scanning. The optimized STIP3–29 showed significant promise as an antimicrobial agent against *MRSA* in a skin-simulated model. Alanine scanning of aurein 1.2, a 13-residue AMP from Australian bell frogs, revealed that replacing Asp4 and Glu11 residues enhanced activity against nearly all tested bacteria (Migoń et al., 2019). Its MIC is 2 μ g/mL, the frequency of drug-resistant mutations is less than 5.0×10^-9^, and the hemolysis rate is only 2.5% (Migoń et al., 2019).

### 5.4 C-terminal amidation and N-terminal acetylation

C-terminal amidation, N-terminal acetylation, or a combination of both can enhance the proteolytic stability of natural and synthetic peptides. Dandan Li computationally designed AMP L163, which was susceptible to trypsin in plasma. Stable L163 analogs were generated via AA substitution, N-terminal acetylation, and cyclization. Acetylation reduced antifungal activity but enhanced antibacterial activity. Acetylation reduced its antifungal activity against Candida albicans from 4 μ M to 8 μ M, but enhanced its antibacterial activity (MIC against Escherichia coli decreased from 8 μ M to 4 μ M). At the same time, it enhanced its stability to temperature and pH changes, and significantly improved its ability to resist trypsin degradation (plasma half-life extended to 12 hours) (Li et al., 2021).

C-terminal amidation of CM15, a short 15-residue synthetic AMP, promotes faster adsorption onto the membrane surface and more efficient disruption of the outer layer, enhancing antimicrobial efficacy (Ma et al., 2021). Studies on amurin-9KY and its derivatives demonstrated that C-terminal amidation was indispensable for antibacterial activity while also contributing to low hemolytic activity (Zhang et al., 2019). Although *in vivo* NMR is beneficial for studying live bacterial cells, its high background noise and low sensitivity pose challenges for directly characterizing AMP conformations. C-terminal amidation is considered a useful tool for overcoming these limitations in probing peptide-lipid interactions via *in vitro* NMR spectroscopy (Zhu et al., 2021).

### 5.5 Cyclisation

Cyclization is prevalent in natural AMPs, occurring via side chain-backbone, side chain-side chain, backbone-backbone, or head-to-tail linkages. For AMP modifications, cyclization can be achieved post-translationally through peptide, lactam, or disulfide bonds. Ultrashort cationic lipopeptides (USCLs) exhibit potent antimicrobial activity but often suffer from high cytotoxicity, limiting their application. Damian Neubauer et al. investigated the bioactivity of N-palmitoylated USCLs after cyclizing their polar heads with disulfide bridges. The results showed that the modified cyclic USCLs significantly improved the selectivity of Gram positive bacteria and Candida (MIC of 1 μ M for MRSA and Candida albicans), which was superior to the linear control (MIC of 8 μ M for MRSA), and the hemolysis rate was reduced to<5% at a concentration of 2 × MIC (Neubauer et al., 2020).

KR-12, an active fragment of LL-37, was engineered by Sunithi Gunasekera via backbone cyclization. A cyclic dimer exhibited a 16-fold increase in antimicrobial activity against *Staphylococcus aureus* and *Pseudomonas aeruginosa*, and an 8-fold increase in fungicidal activity against *Candida albicans*, though hemolysis and cytotoxicity also increased (Gunasekera et al., 2020). A novel bicyclic approach involved cross-linking the ϵ-amino groups of three lysine residues in the cationic AMP OH-CM6 using a 1,3,5-trimethylene benzene spacer. One of the four resulting bicyclic peptides displayed strong antibacterial activity, low cytotoxicity, and enhanced serum stability (He et al., 2022).

### 5.6 PEGylation

Polyethylene glycol (PEG) units are commonly used to improve the half-life, solubility, and pharmacokinetics of peptide drugs. However, coupling PEG moieties to AMPs remains relatively unexplored, despite evidence suggesting PEGylation can reduce cytotoxicity and hemolysis (Imura et al., 2007a; Imura et al., 2007b; Singh et al., 2014). Valerie Ortiz-Gomez and colleagues modified MH5C, a cationic hydrophobic isoform of MH5, by adding a C-terminal cysteine for PEG linkage, providing a protective surface layer. The peptide segment coupled with 5 kDa PEG can effectively clear and inhibit biofilms (with a clearance rate of 89% against Pseudomonas aeruginosa biofilm at a concentration of 8 μ M), significantly higher than free MH5C (with a clearance rate of 35% at the same concentration). The specific mechanism remains to be elucidated (Ortiz-Gómez et al., 2020). Incorporating a cysteine residue at the C-terminus (73c) of a shortened aurein 2.2 allowed covalent attachment to polymers like PEG. Peptides conjugated to PEG-phospholipid micelles showed reduced cytotoxicity against host cells and decreased aggregation (Kumar et al., 2019).

### 5.7 Lipidation

Evidence suggests lipidation can significantly enhance AMP activity. The proline-rich AMP Bac7(1-16) exhibits narrow-spectrum activity against some *Gram-negative bacteria* by inhibiting bacterial protein synthesis (Benincasa et al., 2004; Benincasa et al., 2010; Dolzani et al., 2019; Mardirossian et al., 2020). FFederica Armas modified its C-terminus with a C12-alkyl chain or the cationic lipid Lp-I. A remarkable decrease in MIC and broadened antibacterial spectrum were observed, indicating that C-terminal lipidation profoundly alters the activity and mechanism of ultrashort proline-rich peptides (Armas et al., 2021). Paulina Kosikowska-Adamus et al. performed N-terminal lipidation of DK5 with carnosine derivatives. Modifying the structures of CARPEG-DK5, CAR, and DK5 by lipidating the N-terminus with various carboxylic acids enhanced membrane anchoring properties, leading to improved antimicrobial activity (Kosikowska-Adamus et al., 2021).

## 6 Strategies of targeted delivery

Although antimicrobial peptides have potential in combating drug resistance, their systemic use as drugs requires sufficient circulation time and low cytotoxicity. The combination of targeted delivery strategies and biomaterial delivery systems provides a solution to these problems through precise targeting, carrier protection, and controllable drug release. This chapter integrates targeted delivery strategies and biomaterial carrier technologies, and constructs a systematic AMP delivery system from dimensions such as targeting mechanisms, carrier types, and efficacy optimization.

### 6.1 Targeted delivery core strategy

The core of targeted delivery is to achieve precise enrichment of AMP towards infected sites or target cells. Its strategy can be divided into active targeting and passive targeting, both of which often work synergistically in combination with biomaterial carriers.

Active targeting strategies are mainly divided into two types: pH responsive targeting and receptor-mediated targeting. PH responsive targeting utilizes the acidic microenvironment (pH 5.0~7.0) at the site of infection to achieve targeted release of AMP. Receptor mediated targeting utilizes AMP or carrier surface modified targeting ligands to bind with pathogen/target cell surface receptors for precise delivery. If the LL-37 mutant peptide CKR12 is modified on the surface of polylactic acid glycolic acid copolymer (PLGA) micelles, it can target the surface receptors of Candida albicans, reducing the minimum inhibitory concentration (MIC) of miconazole (MCZ) from 24.03 μ M to 0.24 μ M, achieving precise treatment of fungal infections (Mori et al., 2021).

The passive targeting strategy utilizes the increased vascular permeability at the site of infection, enhancing the permeability and retention (EPR) effect to achieve passive enrichment and promote the retention of the carrier in the lesion. 5.2.2 Biological Carrier Materials and Efficacy.

Biomaterial carriers are the physical and chemical basis for targeted strategies, and the structural characteristics of different carriers determine their targeting efficacy and drug release behavior. They are mainly divided into three categories: inorganic nanocarriers, intelligent responsive carriers, and polymer carriers.

Intracellular infection is caused by bacterial replication within host cells (such as monocytes), which allows bacteria to evade host defense mechanisms. Antibiotics are difficult to penetrate into infected cells, and the global rise in drug resistance makes the treatment of such infections increasingly challenging. Leishmania disease is a typical example, where *Leishmania* parasites parasitize the phagolysosomes of mammalian macrophages without flagella. To solve this problem, Zaofeng Yang et al. utilized submicron Cry3Aa crystals as inorganic nanocarriers: these crystals can be stably and specifically taken up by macrophages and target lysosomes. They used the negative charge region in crystal structure domain II to encapsulate cationic anti *Leishmania* antimicrobial peptide DS1. After a period of treatment, the *in vitro* IC_50_of DS1 against *Leishmania* parasites decreased from>20 μ M to 0.30 μ M. After *in vivo* treatment, the parasitic load of mouse lesions decreased by 42%, and there was no significant hemolysis or hepatotoxicity at a dose of 50 mg/kg. These results indicate that the delivery system can enhance the therapeutic effect on intracellular infections both *in vitro* and *in vivo* (Yang et al., 2019).

PH responsive nanomaterials are excellent carriers of antimicrobial peptides, which can protect them from protein hydrolysis degradation, target infected sites with abnormal pH, and reduce off target effects. Belongs to intelligent responsive carriers. Mark Gontsarik et al. designed and characterized pH responsive nanocarriers based on 1,2-dioloyl-3-dimethylaminopropane (DODAP) self-assembly, and combined them with LL-37. They elucidated the effect of DODAP nanostructures on the encapsulation and release of LL-37, providing ideas for the future development of targeted delivery systems for cationic antimicrobial peptides to anionic bacterial membranes (Gontsarik et al., 2021).

**Figure 3:**
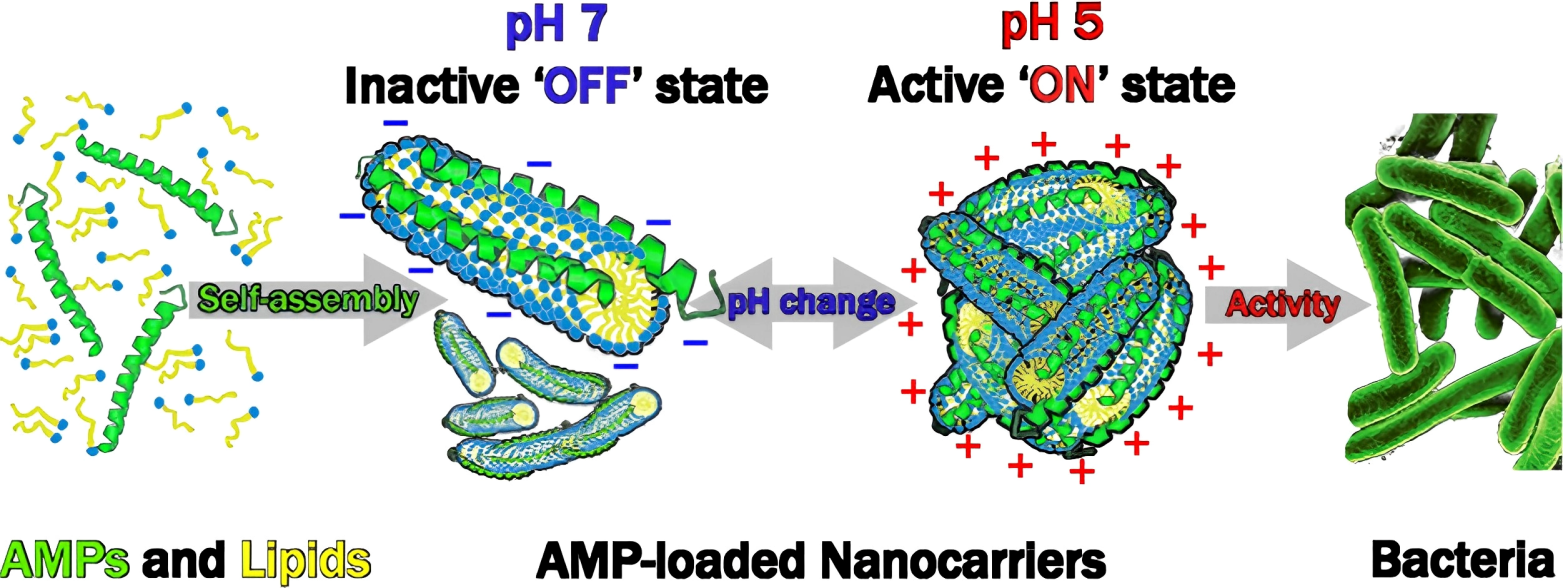
Schematic depicting a design approach for pH-responsive antimicrobial nanomaterial. Figure reproduced from Mark Gontsarik and co-workers after copyright permission (Gontsarik et al., 2018).

In addition, they also designed a pH sensitive antibacterial nanomaterial based on self-assembly of oleic acid (OA) and LL-37, as shown in **Figure 3**. Research has found that under pH 5.0 conditions, positively charged LL-37/OA aggregates exhibit strong antibacterial activity against Escherichia coli and are not affected by other pH conditions; On the contrary, the negatively charged cylindrical micelles formed under pH 7.0 have almost no antibacterial activity. This pH dependent ‘switching’ ability enables antimicrobial peptides to aggregate at the site of infection (Gontsarik et al., 2018).

Polymer polymer carriers have become another important type of carrier for delivering AMPs due to their excellent biocompatibility, degradability, and drug loading properties. The targeted peptide antimicrobial peptide coupled micelles constructed through polymer self-assembly exhibited excellent delivery efficiency. Researchers used a peptide (GSRSKGT) targeting red blood cells infected with Plasmodium falciparum to loosely modify the alpha chain end, while coupling the cyclic decapeptide of the short peptide family to the omega chain end through thiol ene Michael addition reaction. The self-assembled micelle nano aggregates formed by these synthesized structures were characterized using transmission electron microscopy (TEM) and dynamic light scattering (DLS) techniques (Jokonya et al., 2020). The results indicate that the selectivity and molecular weight of these conjugates are improved, and optimizing ligand density can enhance their activity, reduce cytotoxicity, and improve selectivity.

In addition, PLGA carriers also have good biodegradability and drug loading capacity. Its hydrophobic core can encapsulate hydrophobic AMPs, and its hydrophilic shell can prolong the circulation time in the body. CKR12 is a peptide derived from LL-37, which has a conserved mechanism of action and antibacterial activity. Researchers used it to functionalize polylactic acid hydroxyacetic acid copolymer (PLGA) micelles encapsulating the broad-spectrum antifungal drug miconazole (MCZ). Its critical micelle concentration is 12 μ M, drug loading is 23.37%, encapsulation efficiency is 96.85%, and 24-hour cumulative release rate is 52.90%. It can destroy fungal cell walls and membranes to achieve synergistic sterilization, and the structure remains stable even after 24 fold dilution. The results indicate that CKR12 surface modification endows micelle bacteria with targeting ability, significantly enhancing the delivery efficiency of miconazole (Mori et al., 2021).

**Figure 4:**
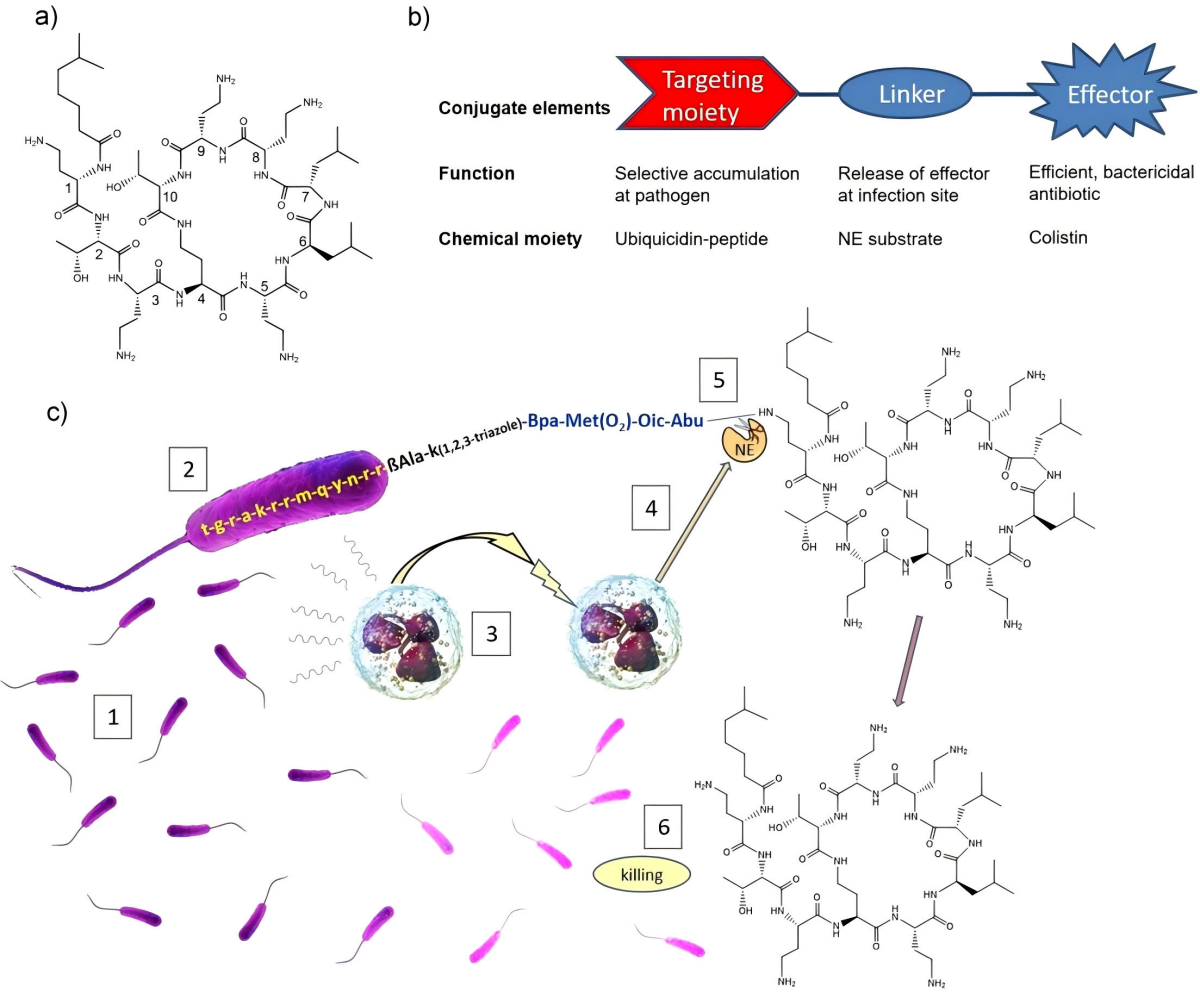
Overview of antibiotic release triggered by selective targeting and infection-triggered. **(a)** the structure of colistin, Numbers refer to the position of amino acids. **(b)** Overview of the conjugate design schematic. **(c)** Mechanism of cellular action. Figure reproduced from Werner Tegge and co-workers after copyright permission (Tegge et al., 2021).

Using AMPs as targeting ligands is another innovative strategy. Werner Tegge et al. utilized modified pan fungicide fragments to achieve bacterial targeting and validated it through fluorescent labeling of variants. They further designed a linker that can be cleaved by neutrophil elastase (NE) and coupled it to the 1st or 3rd amino acid of polymyxin. This design enables the drug to achieve infection responsive release through the secretion of neutrophil elastase in neutrophil rich infection sites (**Figure 4**) (Tegge et al., 2021).

### 6.2 Synergistic effect of antimicrobial peptides and antibiotics

In the increasingly severe clinical context of multidrug-resistant bacterial infections, the therapeutic effect of single antibiotics is gradually limited. The synergistic combination strategy of AMPs and traditional antibiotics, with their complementary mechanisms of action and ability to reduce the risk of drug resistance, has become the core solution to improve the efficacy of anti-infection treatment. This strategy synergizes the membrane targeting properties of AMPs with the intracellular targeting of antibiotics, enhancing bactericidal efficacy while reducing drug dosage and toxicity, providing a new pathway for the treatment of drug-resistant bacterial infections.

The cationic properties and amphiphilic structure of AMP enable it to specifically target bacterial cell membranes, disrupt membrane integrity, create channels for antibiotics to enter the cell, and achieve synergistic sterilization. When levofloxacin and cyclic (R4W4) (a synthetic amphiphilic antimicrobial peptide containing 4 arginine (R) and 4 tryptophan (W) residues) form a physical mixture, cyclic (R4W4) can open up a pathway for levofloxacin to enter bacteria through membrane targeting. The two exhibit significant synergistic antibacterial activity against multiple pathogens, and the cytotoxicity of this combination is extremely low. The core of its synergistic effect lies in the complementary effect of AMP’s targeted destruction of membrane phospholipids and the intracellular antibacterial effect of antibiotics (Sajid et al., 2022).

**Figure 5:**
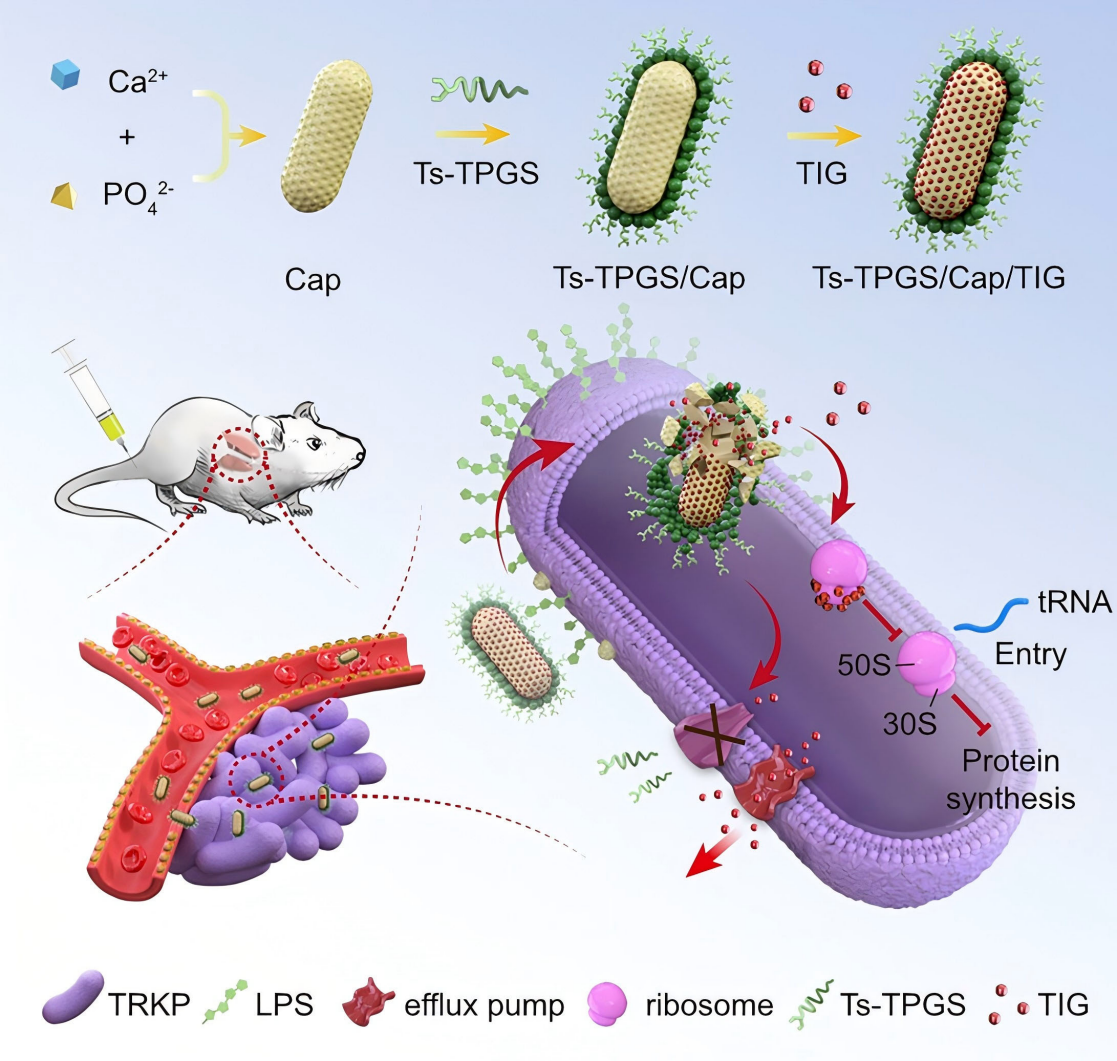
The fabrication of D-alpha tocopheryl polyethylene glycol succinate (TPGS) based and S-thanatin functionalized nanorods. Figure reproduced from Xiaojuan Wang and co-workers after copyright permission (Wang et al., 2022).

The construction of a targeted delivery system further enhances the synergistic effect of AMP and antibiotics in response to the resistance of multidrug-resistant *Klebsiella pneumoniae* to tigecycline. Xiaojuan Wang et al. validated S-sericin functionalized nanorods based on D - α - tocopherol polyethylene glycol succinate (TPGS). These nanorods achieve efficient delivery of tigecycline through a dual mechanism. Firstly, S-sericin peptide can specifically bind to lipopolysaccharides (LPS) on the surface of Gram negative bacteria, achieving bacterial targeted enrichment of drugs; Secondly, TPGS can inhibit the activity of bacterial efflux pump (AcrAB TolC) and downregulate the expression of its coding genes (acrA, acrB) and regulatory gene ramA, reducing the extracellular pumping of tigecycline. This mechanism not only enhances the accumulation of tigecycline in bacteria, but also produces additional synergistic antibacterial effects (**Figure 5**) (Wang et al., 2022).

The study also found that antimicrobial peptides rich in tryptophan exhibit synergistic antibacterial activity against multidrug-resistant Staphylococcus epidermidis (MRSE) when used in combination with traditional antibiotics such as penicillin, ampicillin, and erythromycin. This combination significantly inhibits the formation of MRSE 1208 biofilm by disrupting the bacterial bilayer membrane or downregulating resistance genes to increase intracellular antibiotic concentration (Shang et al., 2019).

### 6.3 Anti-biofilm effect

Bacterial biofilm is a bacterial community enclosed by extracellular matrix, and its formation is one of the key reasons for bacterial resistance. More than 65% of clinical infections are related to biofilm (Shang et al., 2019; Guo et al., 2021). Antimicrobial peptides have become core candidate drugs for anti-biofilm therapy due to their unique membrane activity and matrix penetration ability. It mainly blocks the initial formation of biofilms by interfering with the bacterial quorum sensing (QS) system, inhibiting the expression of adhesion related proteins, and blocking early adhesion processes. A typical example is the alpha - (4-pentenyl) - alanine modified analog K59 of Feleucin-K3, which can significantly downregulate the expression of core genes (lasI, rhlI) in the QS system of *Pseudomonas aeruginosa*, inhibit the synthesis of quorum sensing signaling molecules, and reduce the expression level of pilA, thereby weakening the initial adhesion ability of bacteria. Laser confocal microscopy (CLSM) data showed that 4 μ g/mL of K59 reduced the proportion of viable bacteria in the biofilm of Acinetobacter baumannii ATCC 19606 from 85% in the control group to 30%, and reduced the biofilm thickness by 52% (Guo et al., 2021).

The anti biofilm activity of natural antimicrobial peptides is limited by their matrix penetration ability and protease sensitivity, and can be enhanced through targeted chemical modifications. For example, after N-terminal acetylation modification, the anti trypsin degradation ability of L163 is significantly improved, and 65% of its anti biofilm activity is still retained after 12 hours of plasma incubation, far exceeding the 12% of natural L163 (Li et al., 2021). Bac7 (1-16) undergoes C12 alkyl chain lipidation modification, and its hydrophobic alkyl chain can enhance hydrophobic binding with the extracellular matrix (EPS) of the biofilm and improve membrane insertion efficiency. The clearance rate of carbapenem resistant *Escherichia coli* (CRE) biofilm reaches 78%, and the frequency of drug-resistant mutations is less than 2.0 × 10^-9^ (Armas et al., 2021).

## 7 Conclusions and prospects

AMPs, membrane-active peptides integral to the innate immune systems of multicellular eukaryotes, constitute the first line of host defense. Amidst escalating global antibiotic resistance threats, their potent antimicrobial properties and low resistance development potential have positioned them as promising alternatives to conventional antibiotics. Since the mid-1990s, thousands of AMPs and analogs have been identified, with numerous candidates advancing to clinical trials. However, despite compelling *in vitro* and *in vivo* efficacy, only seven AMPs have received FDA approval for clinical use. Clinical translation remains impeded by inherent limitations: susceptibility to proteolytic degradation, poor oral bioavailability, potential mammalian cytotoxicity, suboptimal *in vivo* efficacy, and prohibitive production costs. Consequently, optimizing synthesis methodologies and delivery strategies to overcome these constraints is paramount for realizing the therapeutic potential of AMPs in clinical anti-infective therapy. A comprehensive understanding of​structure-activity relationships (SAR) is foundational. Most AMPs and analogs exert bactericidal effects through cationic amphiphilic structures that disrupt bacterial membranes or biofilms. However, facially amphiphilic (FA) configurations may concurrently damage eukaryotic membranes, causing cytotoxicity. Furthermore, exposed FA surfaces promote non-specific protein binding and self-aggregation, potentially diminishing antibacterial activity (Chen et al., 2005). SAR insights enable rational modification of natural AMPs or *de novo* design of synthetic analogs to enhance antibiotic properties, targeting precision, or novel functionalities.

This review has examined AMP mechanisms through four classical membrane-disruption models, highlighted recent advances in chemical synthesis/modification techniques, and critically assessed targeted delivery platforms. As demonstrated, SAR-guided chemical design and delivery innovations have achieved substantial progress in overcoming intrinsic AMP limitations – reducing cytotoxicity while enhancing antimicrobial potency, stability, and targeting specificity. The progression of AMPs into frontline clinical therapeutics demands interdisciplinary collaboration spanning microbiology, preclinical research, medicinal chemistry, synthetic chemistry, and pharmaceutics. With continued advancements across these domains, the clinical realization of AMP-based antibacterial therapies appears increasingly attainable in the foreseeable future.
